# A Randomized Controlled Pilot Trial Evaluating the Efficacy of Intravaginal and Extravaginal K-Laser Therapy as a Personalized Non-Hormonal Treatment for Genitourinary Syndrome of Menopause

**DOI:** 10.3390/jpm16070378

**Published:** 2026-07-15

**Authors:** Rocío Martín-Valero, Antonia M. Ruiz-Moreno, Pablo J. Gallardo-García, María Dolores Martínez Colmena, Catalina Muñoz Pagan, Pedro González-Rojas, Paloma Ortega Quiñonero

**Affiliations:** 1 Department of Physiotherapy, Faculty of Health Sciences, CTS-1071 Research Group, University of Malaga, Arquitecto Francisco Peñalosa 3, Ampliación Campus de Teatinos, 29071 Malaga, Spain; pedrogonro2004@gmail.com; 2 Fundación Juan Cruzado, Calle Paganini, 7, Bailén-Miraflores, 29010 Malaga, Spain; antoniaruizmoreno6@gmail.com; 3 Department of Psychology, University of Malaga, 29071 Malaga, Spain; pablojgallardo4@gmail.com; 4 Department of Obstetrics and Gynecology, Santa Lucía General University Hospital, Minarete Street, 30202 Cartagena, Spain; mdmartinezcolmena12@gmail.com (M.D.M.C.); catimpagan76@gmail.com (C.M.P.); palogine@gmail.com (P.O.Q.)

**Keywords:** genitourinary syndrome of menopause, intravaginal laser, K-Laser, pelvic floor dysfunction, Female Sexual Function Index, non-hormonal therapy

## Abstract

**Background/Objectives**: Genitourinary Syndrome of Menopause (GSM) negatively affects quality of life in postmenopausal women, causing sexual dysfunction, vaginal atrophy, and pelvic discomfort. Personalized medicine highlights the need for individualized, non-hormonal therapeutic options for women with contraindications to or preferences against hormonal treatment. Non-hormonal therapies, such as laser treatments, have emerged as potential alternatives, but evidence comparing intravaginal and extravaginal K-Laser therapy remains limited. This study aimed to evaluate the efficacy of intravaginal and extravaginal K-Laser therapy on the symptoms of Genitourinary Syndrome of Menopause (GSM) in postmenopausal women. **Methods**: In this single-center, randomized, single-blind, placebo-controlled trial, 57 postmenopausal women were randomly assigned to receive either intravaginal and extravaginal K-Laser Cube Plus 30 therapy (*n* = 36) or a simulated control treatment (*n* = 21). The primary outcome was sexual function, measured by the Female Sexual Function Index (FSFI). Secondary outcomes included vaginal pH and pelvic floor muscle function assessed via the PERFECT protocol. Outcomes were assessed at baseline and after 6 weeks. **Results**: Sixty-seven women were enrolled, and ten were lost to follow-up. The treatment group showed significant improvements over the control group in FSFI (mean difference = 6.38; *p* < 0.001), PERFECT protocol scores (mean difference = 0.78; *p* = 0.004), CPPQ-Mohedo (mean difference = 5.44; *p* < 0.001), and Menopause Rating Scale (mean difference = 6.50; *p* = 0.017). Significant reductions were also observed in vaginal dryness, vulvar dystrophy, and atrophy (*p* < 0.001). **Conclusions**: Intravaginal and extravaginal K-Laser therapy appears to be a safe and effective non-hormonal treatment for GSM and may support personalized management strategies. However, further well-designed randomized clinical trials with larger samples, longer follow-up of both laser-treated and control groups, and objective outcome measures are needed to provide higher-quality evidence regarding efficacy and identify the patients most likely to benefit.

## 1. Introduction

Sexual health is a significant aspect throughout all stages of a woman’s development, interacting with multiple facets of physical, psychological, emotional, and social well-being. Notably, during the climacteric phase, women experience considerable changes influenced by hormonal fluctuations, which negatively impact their quality of life [1].

Menopause is characterized by hormonal changes, particularly a decline in estrogen levels, which primarily affect the genitourinary system. This hypoestrogenism leads to characteristic symptoms, with vulvovaginal atrophy being highly prevalent. The genitourinary syndrome of menopause (GSM) is a chronic and progressive condition involving vulvovaginal, sexual, and urinary symptoms. Common manifestations include vaginal dryness, itching, burning, irritation, as well as dyspareunia and sexual dysfunction. Urinary symptoms such as incontinence, dysuria, and urinary tract infections are also commonly associated [1].

Vulvovaginal atrophy affects most peri- and postmenopausal women, with prevalence ranging from 36% to 90%. Recent data suggest that this condition can also occur during premenopause, affecting about 19% of women aged 40 to 45 years [2]. The definition and classification of these symptoms have evolved, and in 2014, the International Society for the Study of Women’s Sexual Health (ISSWSH) recommended the term Genitourinary Syndrome of Menopause to better encompass these symptoms instead of vaginal atrophy [3].

Another debilitating symptom affecting quality of life in women with GSM is chronic pelvic pain during urination, defecation, or penetrative sexual intercourse, according to the European Association of Urology (EAU) [4].

Diagnosis of GSM symptoms requires a clinical evaluation including patient history, symptom assessment, and physical examination. Laboratory tests such as vaginal pH measurement and the Vaginal Maturation Index (VMI) may be conducted. Various treatments exist, ranging from systemic to local therapies, including hormonal treatments, lubricants, probiotics, and oral phytoestrogens, depending on whether symptoms are solely vaginal or accompanied by systemic symptoms like hot flashes [5]. Given the heterogeneity of symptom severity, comorbidities, contraindications to hormone therapy, and patient preferences, the management of GSM increasingly requires an individualized approach. In this context, personalized medicine seeks to tailor treatment strategies to the specific clinical characteristics and needs of each woman, highlighting the importance of expanding the range of effective non-hormonal therapeutic options [6].

Regarding Erbium laser application, a 2020 systematic review examined its effects on skin and vaginal walls related to rejuvenation and cosmetic outcomes [7]. The review included 15 studies (11 on skin, 4 on vaginal tissue, including pelvic floor and vaginal wall) involving human tissue samples, rat models, and clinical cases. Despite limited study quality and heterogeneous treatment protocols, consistent effects were reported, including immediate local temperature increase and enhanced coagulation of extracellular matrix in epithelial and subepithelial layers, followed by epithelial thickening, inflammatory response, fibroblast proliferation, increased vascularization, and collagen deposition. Crucially, Er:YAG laser produces these effects without epithelial ablation, promoting cellular activation, extracellular matrix production, and tissue remodeling [7]. Recently, there has been growing interest in K-Laser Cube Plus 30 [8] therapies as a treatment for GSM symptoms. Light Amplification by Stimulated Emission of Radiation (LASER) has been used for over 40 years in gynecology and urology. Clinically, it has been successfully applied for tissue remodeling of non-mucosal tissues, scars, and wrinkles [9], which has facilitated its implementation for treating vaginal atrophy and led to its commercialization as vaginal rejuvenation therapy [7,10]. Additionally, numerous studies report significant improvements in urinary incontinence following laser therapy [11,12,13,14,15]. However, there are few studies on the effectiveness of laser therapy as a treatment with K-Laser Cube Plus 30 for GSM symptoms. Furthermore, evidence regarding its potential contribution to individualized, non-hormonal management strategies remains scarce. Therefore, the objective of this study was to analyze the effectiveness of laser treatment for GSM symptoms, providing evidence that may support personalized therapeutic decision-making in postmenopausal women with GSM.

## 2. Materials and Methods

### 2.1. Study Design

This single-center, randomized, single-blind, placebo-controlled trial (ClinicalTrials.gov ID: NCT05305209) was conducted at the Santa Lucía General University Hospital (Murcia, Spain). The reporting of this randomized controlled trial follows the Consolidated Standards of Reporting Trials (CONSORT) reporting guideline (Supplementary File S1) [16].

### 2.2. Participants

57 postmenopausal women (≥5 years without menstruation) meeting inclusion criteria and providing informed consent. Participants were recruited from November 2023 to May 2025 in Santa Lucía General University Hospital, Murcia, Spain.

### 2.3. Sample Size

The sample size of 70 participants was determined based on feasibility considerations, based on the number of eligible postmenopausal women attending the recruiting hospital during the study period. This pragmatic approach was considered appropriate given the exploratory nature of the trial.

### 2.4. Randomization and Blinding

This was a single-blind randomized controlled trial. Participants were blinded to their group assignment, but the physiotherapists knew the group allocations. Data were entered into an Excel spreadsheet that included the group assignment. The intervention was delivered by a physiotherapist. Outcome assessments were performed by a gynecologist who was also blinded to group allocation. Furthermore, the researcher responsible for data analysis was blinded to group allocation and conducted the statistical analyses without access to the group information. Randomization was conducted by gynecologists during recruitment using the randomization function in Microsoft Excel, and participants were assigned to intervention or control groups. Data were entered without direct access to group allocation by the researcher performing the data analysis. Group assignments were recorded in an Excel sheet into two arms:

Group 1 (Intervention, *n* = 37): 12 sessions of intravaginal and extravaginal K-Laser treatment over 6 weeks.

Group 2 (Control, *n* = 30): Simulated laser therapy under identical conditions.

### 2.5. Intervention

- Intervention group: The patients received 6:30 min of intravaginal application with an average power of 4 watts, with peak power of 8 watts and total applied energy of 1600 J, and an extracavitary application for 3:30 min with an average power of 3 watts and a total dose of 1600 J, with all effects at 200%, twice a week for 6 weeks.
- Control Group: The patients received 6:30 min of intravaginal application and 3:30 min of extracavitary application twice a week for 6 weeks. The difference compared to the intervention of group 1 is that the device remained switched off and did not deliver energy.

The simulated treatment (laser off) was chosen as the comparator to control for expectation bias and the placebo effect, ensuring that participants were unaware of whether they received the active treatment or placebo. Although the study was single-blind because the operator knew the device status, the simulated comparator isolated the specific effect of the diode laser. Therefore, any observed differences were more likely attributable to the intervention rather than to psychological factors or patient expectations.

To preserve blinding, participants were not informed of their group allocation during the study. However, for ethical reasons, after completion of the treatment period and data collection, patients who had been assigned to the control group were invited to receive the full active treatment protocol, ensuring the opportunity to benefit from the expected therapeutic effects.

### 2.6. Ethical Considerations

This study was conducted in accordance with the principles of the Declaration of Helsinki [17]. Ethical approval was obtained from the Malaga Provincial Research Ethics Committee. Written informed consent was obtained from all participants by a member of the research team prior to enrolment. Participants were informed that they could be allocated to either the intervention group or the placebo/control group and provided their consent before participation. No ancillary studies or collection of biological specimens were planned; therefore, no additional consent provisions were required. To protect confidentiality, all personal identifiers were removed from study records, and only coded data were stored, accessible exclusively to the investigators. No specific ancillary or post-trial care was provided. Given the minimal risks associated with the intervention, no compensation plan was established for potential harm resulting from participation.

### 2.7. Eligibility Criteria

Participants were eligible for inclusion if they had been postmenopausal for at least five years, exhibited GSM symptoms (such as vaginal dryness, dyspareunia, or urinary urgency), and had not received hormone therapy for at least four months. Conversely, the exclusion criteria ruled out individuals with pelvic organ prolapse (POP-Q > stage 2), severe urinary or fecal incontinence, active genital infections, vulvodynia, or recent pelvic surgery. Additionally, those with psychiatric or neurological conditions that might hinder protocol adherence were also excluded.

### 2.8. Outcome Measures

#### 2.8.1. Primary Outcomes

Sexual function (FSFI) [18].

A 19-item questionnaire covering six domains—desire, arousal, lubrication, orgasm, satisfaction, and pain—with scores ranging from 2 to 36, where higher scores indicate better sexual function.

#### 2.8.2. Secondary Outcomes

1. Pelvic floor muscle function (PERFECT protocol) [19,20,21].

The PERFECT assessment scheme is a clinical acronym used to evaluate pelvic floor muscle contractility through digital palpation and manometry, measuring muscle strength (OXFORD scale), endurance, repetitions, quick contractions, and contraction timing.

2. Menopausal symptoms (Menopause Rating Scale, MRS) [22,23,24].

An 11-item scale rated 0 to 4 that evaluates symptom severity and tracks changes over time, validated in multiple languages.

3. Quality of life (SF-12, Cervantes scale) [25,26,27].

The SF-12 is a shortened version of the SF-36 with 12 items assessing physical and mental health components. Reported minimal clinically important difference (MCID) values for the SF-12 vary across studies, ranging from 4.1–11.1 points for the Physical Component Summary (PCS) and 4.7–9.7 points for the Mental Component Summary (MCS), with commonly used thresholds of approximately 8.1 and 4.7 points, respectively [28].

The Cervantes scale is a 31-item tool specifically designed for Spanish women aged 45–64, addressing vasomotor symptoms, sexuality, relationships, and psychological well-being.

4. Pelvic pain (CPPQ-Mohedo) [29].

A 7-item questionnaire used to screen for and monitor chronic pelvic pain.

5. Pelvic floor dysfunction (PFDI-20) [30,31].

A 20-item questionnaire divided into three subscales assessing symptoms related to prolapse, colorectal-anal issues, and urinary problems.

### 2.9. Data Management

All study data were entered into an electronic database with predefined coding. Access to the database was restricted to the investigators handling the data. Double data entry and range checks were performed to ensure data quality.

## 3. Results

### 3.1. Statistical Analysis

The data were analyzed using repeated-measures ANOVA for pre-post comparisons. Missing data were handled by excluding participants who dropped out after the first session. Only data from participants who completed the full intervention were included in the analysis. Post-hoc comparisons were performed to assess group differences at the post-intervention stage. Due to the exploratory nature of the study and the absence of post-treatment data for participants lost to follow-up, a per-protocol analysis was conducted.

### 3.2. Descriptive Statistics

**Figure 1:**
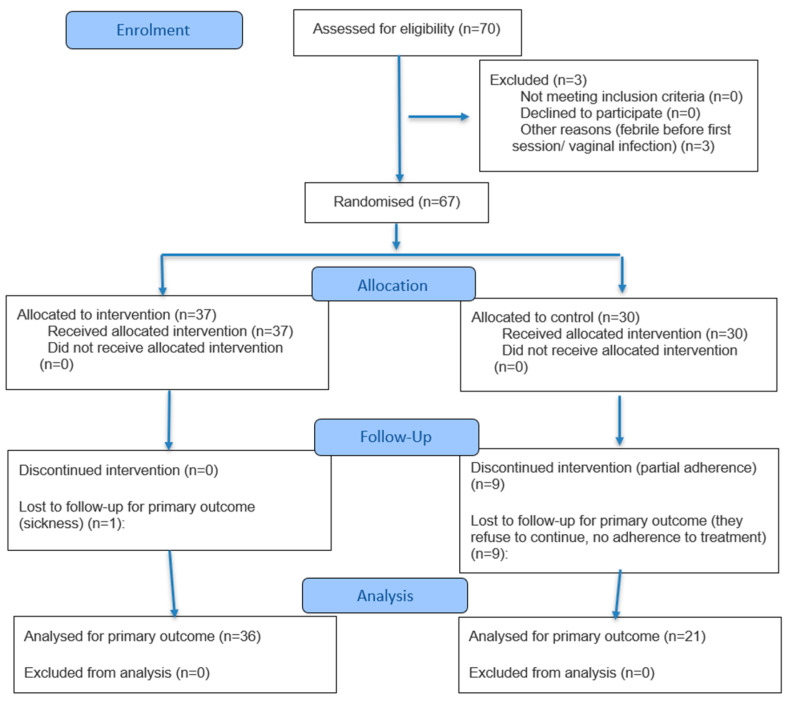
Flow diagram illustrating the recruitment procedure followed in this research.

First, sixty-seven women were enrolled, and ten were lost to follow-up. For each group, the majority of losses and exclusions after randomization were due to illness, including infections accompanied by fever, which prevented participants from continuing the intervention. A detailed flow diagram illustrating participant retention and reasons for exclusion is provided Figure 1. The descriptive statistics are presented in Table 1 and Table 2.

#### 3.2.1. Analysis of General Variance

##### ANOVA: Scale Variables

Table 3 shows the repeated measures ANOVAs with one between-subjects factor performed to examine the components of variability for each of the dependent variables discussed.

Results are from repeated measures ANOVA with intervention as the between-subjects factor. PRE–POST represents the within-subjects factor (time), Intervention represents the between-subjects factor, and PRE–POST × Intervention represents their interaction. η^2^p indicates partial eta squared. FSFI demonstrated significant effects for time (*p* = 0.020; η^2^p = 0.094), group (*p* = 0.002; η^2^p = 0.158), and time × group interaction (*p* = 0.007; η^2^p = 0.125). PERFECT showed the same pattern: time (*p* = 0.036; η^2^p = 0.077), group (*p* = 0.009; η^2^p = 0.117), and interaction (*p* = 0.006; η^2^p = 0.129) were significant.

SF-12 PCS experienced no time effect (*p* = 0.630) but had significant group (*p* = 0.019; η^2^p = 0.096) and interaction effects (*p* < 0.001; η^2^p = 0.292). SF-12 MCS revealed no significant effects, though interaction approached significance (*p* = 0.098; η^2^p = 0.049).

CERVANTES showed no main effects but a significant interaction (*p* < 0.001; η^2^p = 0.224). PFDI-20 showed significant time (*p* = 0.036; η^2^p = 0.078) and interaction effects (*p* = 0.038; η^2^p = 0.076).

MRS demonstrated significant time (*p* = 0.012; η^2^p = 0.110) and strong interaction effects (*p* < 0.001; η^2^p = 0.397). CPPQ-MOHEDO showed significant time (*p* = 0.011; η^2^p = 0.113), group (*p* = 0.022; η^2^p = 0.092), and interaction (*p* < 0.001; η^2^p = 0.373) effects.

##### ANOVA: Gynaecological Variables

The results relating to the gynaecological variables analysed are presented in Table 4.

Regarding pH levels, significant main effects of time (*p* = 0.042; η^2^p = 0.074) and group (*p* = 0.047; η^2^p = 0.071) were found, with no significant interaction. Vaginal dryness showed strong time (*p* < 0.001; η^2^p = 0.472) and smaller group effects (*p* = 0.029; η^2^p = 0.083), but no interaction. Vulvar dystrophy demonstrated significant effects for both time (*p* < 0.001; η^2^p = 0.182) and group (*p* < 0.001; η^2^p = 0.212), without interaction. Vaginitis showed a significant time effect only (*p* < 0.001; η^2^p = 0.267). Vaginal atrophy revealed significant main effects for time (*p* < 0.001; η^2^p = 0.432) and group (*p* = 0.046; η^2^p = 0.070), with no interaction.

##### Post-Hoc Comparisons

Post-hoc comparisons of all scale variables analysed are reported in Table 5.

**Figure 2:**
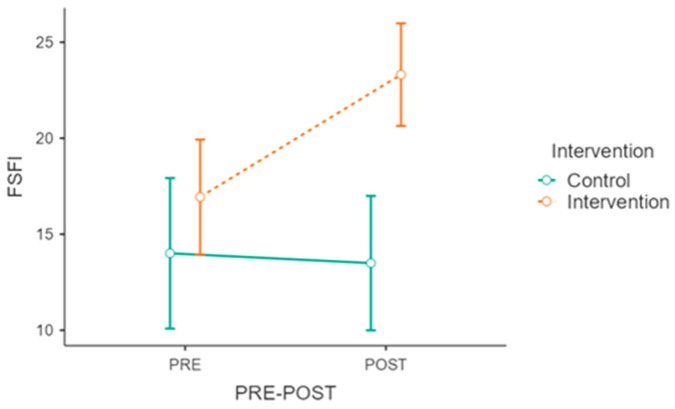
Estimated marginal FSFI averages.

For the FSFI scale, no baseline differences were observed; however, only the intervention group improved significantly after treatment (*p*_Holm_ < 0.001) and scored higher than the control post-intervention (*p*_Holm_ < 0.001) (Figure 2).

**Figure 3:**
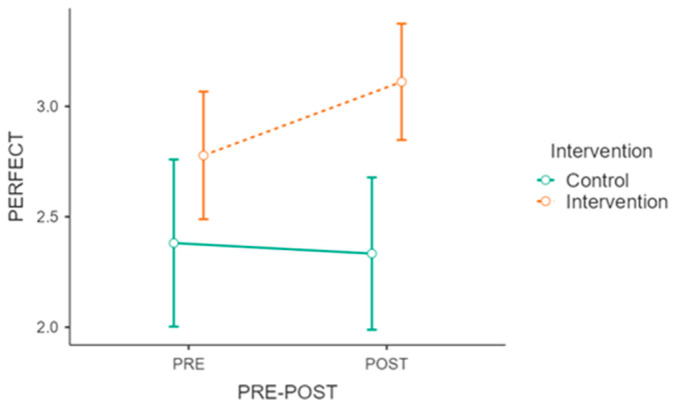
Estimated marginal PERFECT averages.

On the PERFECT scale, a similar pattern is observed, with post-intervention superiority of the intervention group (*p*_Holm_ = 0.004) (Figure 3).

**Figure 4:**
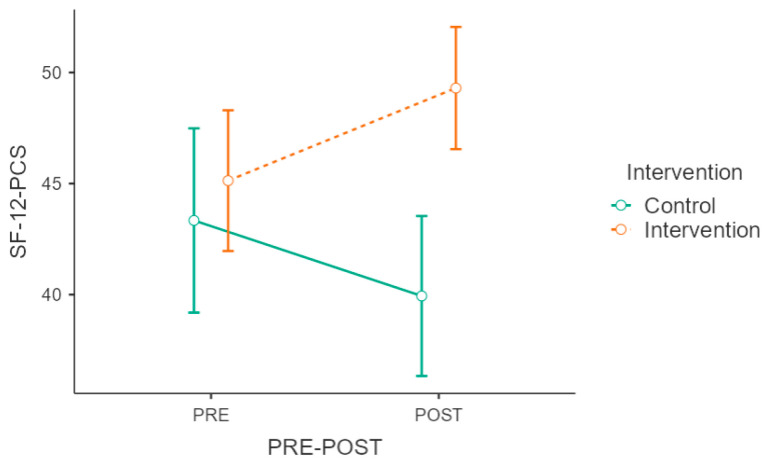
Estimated marginal means of SF-12-PCS.

For the SF-12 PCS P scale, baseline values did not differ. After the intervention, the control group’s scores declined (*p*_Holm_ = 0.038), whereas the intervention group’s scores improved (*p*_Holm_ < 0.001), resulting in a significant post-intervention difference favouring the intervention group (*p*_Holm_ < 0.001). SF-12 MCS showed no significant changes (Figure 4).

CERVANTES scores did not differ at baseline; only the intervention group improved significantly (*p*_Holm_ < 0.001), with no post-intervention difference between groups. On PFDI-20, improvements were found exclusively in the intervention group (*p*_Holm_ = 0.005).

**Figure 5:**
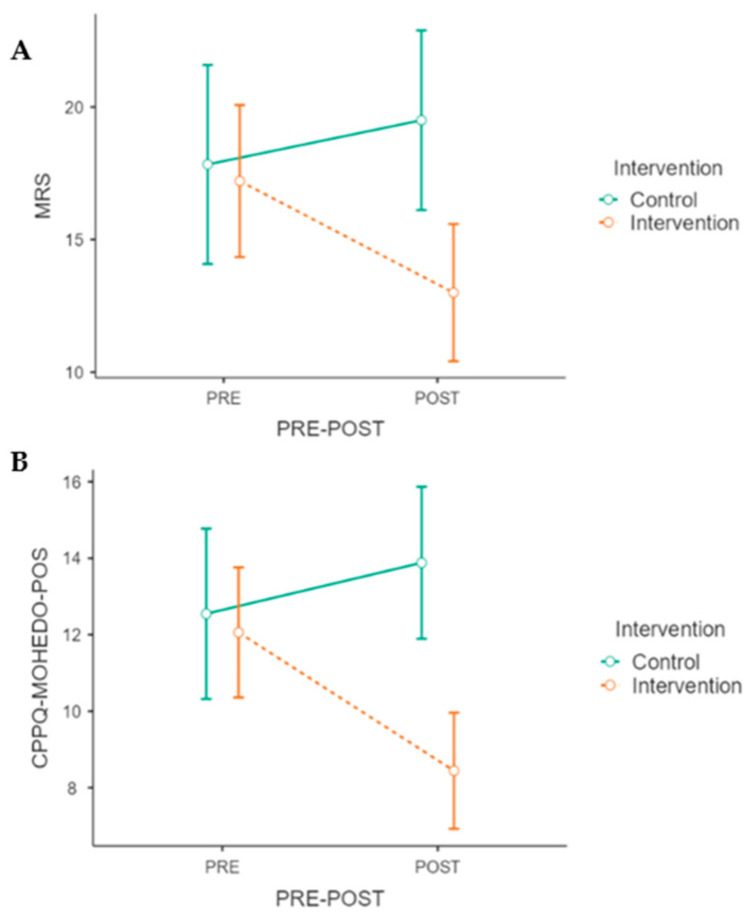
Estimated marginal MRS (**A**) and CPPQ-MOHEDO (**B**).

For the MRS, the intervention group improved (*p*_Holm_ < 0.001) and scored lower than the control post-intervention (*p*_Holm_ = 0.017). CPPQ-MOHEDO showed the same trend (*p*_Holm_ < 0.001) (Figure 5A,B).

For pH, no significant pairwise differences emerged. As detailed in Table 6, both groups improved in dryness, but the intervention group showed greater improvement (*p*_Holm_ = 0.007). In vulvar dystrophy, only the intervention group improved (*p*_Holm_ < 0.001) and achieved lower scores than the control post-intervention (*p*_Holm_ < 0.001). Vaginitis improved only in the intervention group (*p*_Holm_ < 0.001), with no between-group difference. Vaginal atrophy decreased in both groups, with no post-intervention difference.

## 4. Discussion

This study showed significant benefits in female sexual function (FSFI), chronic pelvic pain (CPPQ-Mohedo), and menopausal symptoms (MRS) following intravaginal K-Laser therapy in postmenopausal women with GSM. FSFI scores increased significantly in the intervention group (mean difference = −6.375, SE = 1.489, *p* < 0.001) with no change in controls; CPPQ-Mohedo showed reduced symptom severity (mean difference = 4.103, SE = 1.345, *p* = 0.014), and MRS scores decreased (mean difference = 4.208, SE = 0.593, *p* < 0.001). These findings add to the growing body of evidence supporting non-ablative laser therapies as safe, well-tolerated alternatives for women who cannot or prefer not to use hormonal therapy. From a personalized medicine perspective, these findings reinforce the importance of expanding individualized therapeutic options for women with GSM, as treatment selection should consider not only symptom severity but also clinical characteristics, contraindications, and patient preferences.

In 2007, the American College of Obstetricians and Gynecologists (ACOG) listed “vaginal rejuvenation” and “vaginoplasty” among procedures lacking confirmed safety and efficacy [32]. Subsequently, the FDA approved CO_2_ laser devices in 2010 for various surgical indications, including gynecology [33], with similar approvals for other lasers in 2011–2014 [19,34]. These approvals highlight the popularity of laser devices for vaginal atrophy and gynecological disorders, driving their commercial availability [9].

The most commonly used lasers for GSM symptom treatment are Erbium and CO_2_ lasers. CO_2_ lasers create controlled epithelial micro-injuries to stimulate repair [35].

Ruanphoo et al. (2020) reported significant improvements in VHI, VAS, and dryness after three CO_2_ laser sessions in a randomized trial [36].

Samuels (2018) found positive effects in urinary incontinence, FSFI, and GSM symptoms, with histological evidence of increased collagen and elastin [37].

Er:YAG lasers, reviewed systematically in 2020 [7], increase local temperature and stimulate fibroblast proliferation, vascularization, and collagen deposition without epithelial ablation. However, few studies have rigorously assessed laser efficacy in GSM, with most lacking control groups or randomization [12,38,39].

Concerns about insufficient evidence have been raised by expert groups such as The International Society for the Study of Vulvovaginal Disease (ISSVD) [40] and The Society of Obstetricians and Gynaecologists of Canada (SOGC) [32]. Ablative lasers, although effective, carry risks such as burns and scarring [41].

Despite growing interest in laser therapies for GSM, robust evidence on their efficacy and safety remains limited, particularly regarding novel devices designed to minimize risks associated with conventional ablative lasers. This randomized controlled trial aimed to evaluate the effectiveness of the K-Laser Cube Plus 30 in improving GSM symptoms and pelvic floor dysfunctions. Our study demonstrated significant improvements in sexual function (FSFI) and pelvic floor muscle performance (PERFECT) in the intervention group compared to controls. Additionally, significant positive changes were observed in menopausal symptoms (MRS) and chronic pelvic pain (CPPQ-Mohedo) following treatment. Although favorable changes were also observed in health-related quality of life as measured by the SF-12, the magnitude of change did not reach the commonly reported minimal clinically important difference (MCID) thresholds for either the Physical Component Summary (PCS) or the Mental Component Summary (MCS). Therefore, these findings should be interpreted with caution, as statistical significance may not necessarily translate into clinically meaningful improvements in perceived quality of life. These findings provide new evidence supporting the potential clinical benefits of this innovative laser therapy for women with GSM and related pelvic floor disorders.

Beyond demonstrating clinical efficacy, our findings have implications for personalized medicine. GSM is a heterogeneous condition in which symptom severity, quality-of-life impairment, response to previous treatments, contraindications to hormone therapy, and patient preferences vary considerably. Consequently, therapeutic decisions should be individualized rather than based on a single treatment strategy. In this context, K-Laser therapy may expand the range of evidence-based non-hormonal options available for women who cannot receive estrogen therapy, have experienced insufficient benefit from conventional treatments, or prefer non-hormonal interventions. Rather than replacing established therapies, K-Laser may contribute to a more individualized therapeutic approach within the multidisciplinary management of GSM.

These results are consistent with Seganfredo et al. [42], who reported functional and symptomatic benefits with promestriene, fractional CO_2_ laser, and microablative radiofrequency. Although our intervention using K-Laser differs in type and parameters, the gains align with their reported functional and symptomatic benefits [42].

Furthermore, Salinas Pena et al. [43] showed significant improvements in vaginal maturation value and reduced dyspareunia in women treated with a dual-wavelength laser (1540 nm and 10,600 nm). Although vaginal maturation returned to baseline by 9 months, subjective symptoms remained improved [43]. This underscores the need for longer follow-up, which should be considered in future K-Laser studies.

Unlike ablative lasers such as CO_2_, K-Laser uses a combination of wavelengths in continuous or pulsed mode, enabling deep thermal action with minimal risk of epithelial damage. As described in our study protocol, the aim was to induce a biomodulatory rather than ablative effect, promoting tissue regeneration and improving pelvic floor function without altering superficial vaginal architecture.

The significant improvement observed on the CPPQ-Mohedo scale also suggests potential effects of K-Laser in modulating chronic pelvic pain, an area not widely explored in previous laser studies. This might be related to improved vascularization, reduced inflammation, and tissue regeneration promoted by photobiomodulation, as proposed in other therapeutic laser applications in physical therapy and regenerative medicine.

Clinically, these results have important implications. GSM remains underdiagnosed and undertreated despite its high prevalence and negative impact on quality of life. While topical estrogens remain first-line therapy, poor adherence and contraindications in some groups (e.g., hormone-sensitive cancer survivors) justify the development of non-hormonal alternatives. Within the framework of personalized medicine, the availability of different evidence-based therapeutic options allows clinicians to tailor treatment according to each woman’s clinical characteristics, symptom profile, therapeutic goals, contraindications, and personal preferences. In this context, K-Laser represents a safe, innovative, and non-invasive option suitable for a broad patient profile.

### Study Limitations

However, some limitations must be acknowledged, including the relatively small sample size and short follow-up duration. Outcome assessment was limited to the immediate post-treatment period (6 weeks), which precludes conclusions regarding the long-term durability of the observed benefits. Given that GSM is a chronic and progressive condition, it remains unclear whether the improvements achieved are sustained over time or whether maintenance treatments may be required. Although our results are statistically significant, studies with greater statistical power and longer follow-up are necessary to evaluate the durability of these effects and identify any delayed adverse events. Future research should therefore incorporate extended follow-up periods to better characterize the persistence of treatment effects. In addition, the use of a per-protocol analysis may have introduced attrition bias and potentially overestimated treatment effects. Furthermore, the study was not powered based on a formal sample size calculation; therefore, negative findings should be interpreted cautiously and require confirmation in larger, adequately powered trials. Another limitation is that minimal clinically important difference (MCID) values were only available for the SF-12 outcome. For the remaining outcome measures, no established MCID thresholds were identified in the literature, preventing assessment of whether the observed changes reached a clinically meaningful magnitude from the patient’s perspective. Consequently, although statistically significant improvements were observed, the clinical relevance of these changes could not be fully determined.

In conclusion, our findings strengthen the existing body of evidence supporting energy-based therapies for GSM treatment, particularly K-Laser, for which the literature was previously limited. This study shows that improvements in sexual health, pelvic comfort, and menopausal symptoms can be achieved through a well-tolerated intervention with potential for outpatient use and minimal morbidity. Furthermore, these findings support the integration of K-Laser therapy into individualized management strategies, expanding the therapeutic options available for women requiring personalized, non-hormonal care for GSM. Nevertheless, further well-designed randomized clinical trials involving larger patient populations, longer follow-up of both laser-treated and control groups, and the assessment of objective outcome measures are warranted to provide higher-quality evidence regarding the efficacy and long-term effectiveness of K-Laser therapy for genitourinary syndrome of menopause.

## 5. Conclusions

The findings of this study support the use of intravaginal K-Laser therapy as a safe, effective, and non-hormonal intervention for the treatment of genitourinary syndrome of menopause (GSM) in postmenopausal women. The intervention resulted in clinically relevant improvements in sexual function, chronic pelvic pain, and vasomotor and urogenital symptoms associated with menopause, as measured by validated instruments (FSFI, CPPQ-Mohedo, and MRS). These results contribute to the emerging evidence supporting photobiomodulation as an innovative therapeutic alternative, especially for women with contraindications to or reluctance toward topical estrogen therapies.

Despite methodological limitations, including the sample size and follow-up period, the data obtained allow us to propose K-Laser as a promising therapeutic tool in the multidimensional management of GSM. However, further well-designed, randomized clinical trials with larger sample sizes, longer follow-up of both laser-treated and control groups, and the assessment of objective outcome measures are required to provide the highest-quality evidence regarding the efficacy of K-Laser therapy. Such studies will also help identify the patient profiles most likely to benefit from this intervention and better define its role within personalized treatment strategies for GSM. Future research should focus on assessing the long-term sustainability of its effects, its impact on histological and microbiological parameters, and direct comparisons with other available energy-based technologies.

## Figures and Tables

**Table 1:** Descriptive demographic characteristics for each group and Descriptive Variables Scale.

Scale Variables	Group	N	Mean	SE	SD
FSFI_PRE	Control	21	14.005	2.128	9.753
	Intervention	36	16.939	1.414	8.481
FSFI_POS	Control	21	13.495	1.766	8.093
	Intervention	36	23.314	1.326	7.956
SF_12_PCS_P_PRE	Control	21	43.338	2.385	10.930
	Intervention	36	45.131	1.425	8.553
SF_12_PCS_P_POS	Control	21	39.938	2.063	9.455
	Intervention	36	49.300	1.242	7.451
SF_12_MCS_M_PRE	Control	21	43.410	3.010	13.793
	Intervention	36	45.947	1.867	11.204
SF_12_MCS_M_POS	Control	21	43.324	2.435	11.160
	Intervention	36	49.644	1.508	9.046
CPPQ_MOHEDO_PRE	Control	21	12.548	1.158	5.305
	Intervention	36	12.061	0.828	4.968
CPPQ_MOHEDO_POS	Control	21	13.881	1.080	4.947
	Intervention	36	8.444	0.716	4.297
PFDI_20_PRE	Control	21	69.018	12.221	56.004
	Intervention	36	75.735	9.387	56.324
PFDI_20_POS	Control	21	68.920	11.890	54.487
	Intervention	36	58.565	7.691	46.148
ESCALA_MRS_PRE	Control	21	17.833	2.212	10.136
	Intervention	36	17.208	1.259	7.555
ESCALA_MRS_POS	Control	21	19.500	1.850	8.476
	Intervention	36	13.000	1.216	7.298
CERVANTES_PRE	Control	21	60.190	6.138	28.126
	Intervention	36	62.694	3.366	20.196
CERVANTES_POS	Control	21	64.762	5.923	27.144
	Intervention	36	54.083	3.226	19.356
PERFECT_PRE	Control	21	2.381	0.189	0.865
	Intervention	36	2.778	0.144	0.866
PERFECT_POS	Control	21	2.333	0.199	0.913
	Intervention	36	3.111	0.118	0.708

SE: Standard Error of the Mean. SD: Standard Deviation. POS: Post-Intervention PRE: Pre-Intervention.

**Table 2:** Descriptive gynaecological variables.

Gynaecological Variables	Group	N	Mean	SE	SD
PH_PRE	Control	21	5.976	0.122	0.558
	Intervention	36	5.681	0.134	0.803
PH_POS	Control	20	5.700	0.117	0.523
	Intervention	36	5.428	0.094	0.566
DRYNESS_PRE	Control	21	1.952	0.176	0.805
	Intervention	36	1.806	0.173	1.037
DRYNESS_POS	Control	21	1.190	0.190	0.873
	Intervention	36	0.500	0.116	0.697
VULVA DYSTROPHY_PRE	Control	21	1.714	0.197	0.902
	Intervention	36	1.167	0.171	1.028
VULVA DYSTROPHY_POS	Control	21	1.381	0.234	1.071
	Intervention	36	0.417	0.108	0.649
VAGINITIS_PRE	Control	21	1.143	0.221	1.014
	Intervention	36	1.278	0.185	1.111
VAGINITIS_POS	Control	21	0.667	0.199	0.913
	Intervention	36	0.278	0.094	0.566
VAGINAL ATROPHY_PRE	Control	21	2.143	0.125	0.573
	Intervention	36	1.861	0.133	0.798
VAGINAL ATROPHY_POS	Control	21	1.333	0.187	0.856
	Intervention	36	0.944	0.132	0.791

SE: Standard Error of the Mean. SD: Standard Deviation. POS: Post-Intervention PRE: Pre-Intervention.

**Table 3:** General ANOVA analysis of scale variables.

Scale Variables	Factors	gl	Quadratic Mean	F	*p*	η^2^p
FSFI	PRE-POST	1	228.152	5.715	0.020	0.094
	PRE-POST × Intervention	1	314.315	7.874	0.007	0.125
	Intervention	1	1.078.516	10.317	0.002	0.158
	Residual	55	39.918			
PERFECT	PRE-POST	1	0.541	4.598	0.036	0.077
	PRE-POST × Intervention	1	0.962	8.173	0.006	0.129
	Intervention	1	9.150	7.302	0.009	0.117
	Residual	55	0.118			
SF12PCS_P	PRE-POST	1	3.926	0.234	0.630	0.004
	PRE-POST × Intervention	1	379.966	22.662	<0.001	0.292
	Intervention	1	825.100	5.849	0.019	0.096
	Residual	55	16.766			
SF12MCS_M	PRE-POST	1	86.496	2.575	0.114	0.045
	PRE-POST × Intervention	1	94.902	2.826	0.098	0.049
	Intervention	1	520.380	2.445	0.124	0.043
	Residual	55	33.587			
CERVANTES	PRE-POST	1	108.221	1.495	0.227	0.026
	PRE-POST × Intervention	1	1.152.431	15.918	<0.001	0.224
	Intervention	1	443.150	0.452	0.504	0.008
	Residual	55	72.397			
PFDI_20	PRE-POST	1	1.977.406	4.634	0.036	0.078
	PRE-POST × Intervention	1	1.932.944	4.530	0.038	0.076
	Intervention	1	87.783	0.017	0.897	0.000
	Residual	55	426.697			
ESCALA_MRS	PRE-POST	1	42.840	6.777	0.012	0.110
	PRE-POST × Intervention	1	228.893	36.209	<0.001	0.397
	Intervention	1	336.656	2.643	0.110	0.046
	Residual	55	6.321			
CPPQ_MOHEDO	PRE-POST	1	34.574	6.977	0.011	0.113
	PRE-POST × Intervention	1	162.490	32.789	<0.001	0.373
	Intervention	1	232.650	5.587	0.022	0.092
	Residual	55	4.956			

**Table 4:** Mixed-design ANOVA results for gynaecological variables.

Gynaecological Variables	Factors	gl	Quadratic Mean	F	*p*	η^2^p
PH	PRE-POST	1	1.625	4.323	0.042	0.074
	PRE-POST × Intervention	1	0.000	0.000	0.991	0.000
	Intervention	1	1.886	4.134	0.047	0.071
	Residual	54	0.376			
DRYNESS	PRE-POST	1	28.346	49.143	<0.001	0.472
	PRE-POST × Intervention	1	1.960	3.398	0.071	0.058
	Intervention	1	4.649	5.004	0.029	0.083
	Residual	55	0.577			
VULVA DYSTROPHY	PRE-POST	1	7.783	12.333	<0.001	0.183
	PRE-POST × Intervention	1	1.151	1.824	0.182	0.032
	Intervention	1	15.159	14.814	<0.001	0.212
	Residual	55	0.631			
VAGINITIS	PRE-POST	1	14.451	20.061	<0.001	0.267
	PRE-POST × Intervention	1	1.820	2.526	0.118	0.044
	Intervention	1	0.428	0.452	0.504	0.008
	Residual	55	0.720			
VAGINAL ATROPHY	PRE-POST	1	19.760	41.810	<0.001	0.432
	PRE-POST × Intervention	1	0.076	0.161	0.690	0.003
	Intervention	1	2.983	4.159	0.046	0.070
	Residual	55	0.473			

Results are from repeated measures ANOVA with intervention as the between-subjects factor. PRE–POST represents the within-subjects factor (time), Intervention represents the between-subjects factor, and PRE–POST × Intervention represents their interaction. η^2^p indicates partial eta squared.

**Table 5:** Post-hoc comparisons. Scale variables.

	PRE-POST	Group	PRE-POST	Group	Difference of Means	SE	t	*p*_Holm_
FSFI	PRE	Control	PRE	Intervention	−2.934	2.462	−1.192	0.477
			POST	Control	0.510	1.950	0.261	0.795
		Intervention	POST	Intervention	−6.375	1.489	−4.281	<0.001
	POST	Control	POST	Intervention	−9.819	2.198	−4.467	<0.001
PERFECT	PRE	Control	PRE	Intervention	−0.397	0.238	−1.670	0.201
			POST	Control	0.048	0.106	0.450	0.655
		Intervention	POST	Intervention	−0.333	0.081	−4.121	<0.001
	POST	Control	POST	Intervention	−0.778	0.217	−3.591	0.004
SF12PCS_P	PRE	Control	PRE	Intervention	−1.792	2.605	−0.688	0.494
			POST	Control	3.400	1.264	2.691	0.038
		Intervention	POST	Intervention	−4.169	0.965	−4.320	< 0.001
	POST	Control	POST	Intervention	−9.362	2.262	−4.140	<0.001
SF12MCS_M	PRE	Control	PRE	Intervention	−2.538	3.352	−0.757	1.000
			POST	Control	0.086	1.789	0.048	1.000
		Intervention	POST	Intervention	−3.697	1.366	−2.707	0.054
	POST	Control	POST	Intervention	−6.321	2.710	−2.333	0.117
CERVANTES	PRE	Control	PRE	Intervention	−2.504	6.423	−0.390	1.000
			POST	Control	−4.571	2.626	−1.741	0.436
		Intervention	POST	Intervention	8.611	2.006	4.294	<0.001
	POST	Control	POST	Intervention	10.679	6.179	1.728	0.436
PFDI_20	PRE	Control	PRE	Intervention	−6.717	15.434	−0.435	1.000
			POST	Control	0.098	6.375	0.015	1.000
		Intervention	POST	Intervention	17.170	4.869	3.527	0.005
	POST	Control	POST	Intervention	10.355	13.549	0.764	1.000
ESCALA_MRS	PRE	Control	PRE	Intervention	0.625	2.357	0.265	0.792
			POST	Control	−1.667	0.776	−2.148	0.145
		Intervention	POST	Intervention	4.208	0.593	7.101	<0.001
	POST	Control	POST	Intervention	6.500	2.127	3.056	0.017
CPPQ_MOHEDO	PRE	Control	PRE	Intervention	0.487	1.399	0.348	0.729
			POST	Control	−1.333	0.687	−1.941	0.172
		Intervention	POST	Intervention	4.103	1.345	3.051	0.014
	POST	Control	POST	Intervention	5.437	1.248	4.357	<0.001

Post-hoc pairwise comparisons utilizing Holm’s adjustment method (*p*_Holm_). SE: Standard Error; t: Student’s t-statistic; PRE: Baseline assessment; POST: Post-intervention assessment.

**Table 6:** Post-hoc pairwise comparisons with Holm’s correction for gynaecological variables.

Gynaecological Variables	PRE-POST	Group	PRE-POST	Group	Difference of Means	SE	t	*p*_Holm_
PH	PRE	Control	PRE	Intervention	0.269	0.203	1.329	0.568
			POS	Control	0.250	0.194	1.289	0.568
		Intervention	POS	Intervention	0.253	0.145	1.749	0.411
	POS	Control	POS	Intervention	0.272	0.154	1.771	0.411
DRYNESS	PRE	Control	PRE	Intervention	0.147	0.263	0.558	0.579
			POST	Control	0.762	0.234	3.251	0.007
		Intervention	POST	Intervention	1.306	0.179	7.293	<0.001
	POST	Control	POST	Intervention	0.690	0.210	3.284	0.007
VULVA DYSTROPHY	PRE	Control	PRE	Intervention	0.548	0.270	2.026	0.143
			POST	Control	0.333	0.245	1.360	0.359
		Intervention	POST	Intervention	0.750	0.187	4.006	<0.001
	POST	Control	POST	Intervention	0.964	0.227	4.242	<0.001
VAGINITIS	PRE	Control	PRE	Intervention	−0.135	0.296	−0.456	0.650
			POST	Control	0.476	0.262	1.818	0.155
		Intervention	POST	Intervention	1.000	0.200	4.999	<0.001
	POST	Control	POST	Intervention	0.389	0.196	1.989	0.155
VAGINAL ATROPHY	PRE	Control	PRE	Intervention	0.282	0.199	1.416	0.176
			POST	Control	0.810	0.212	3.816	0.001
		Intervention	POST	Intervention	0.917	0.162	5.657	<0.001
	POST	Control	POST	Intervention	0.389	0.224	1.737	0.176

SE: Standard Error of the Mean. SD: Standard Deviation. POS: Post-Intervention PRE: Pre-Intervention.

## Data Availability

The datasets generated and analyzed during the current study are not publicly available due to privacy restrictions but are available from the corresponding author upon reasonable request.

## Supplementary Materials

The following supporting information can be downloaded at https://www.mdpi.com/article/10.3390/jpm16070378/s1: File S1: CONSORT checklist.

## Abbreviations

The following abbreviations are used in this manuscript:

GSM	Genitourinary Syndrome of Menopause.
FSFI	Female Sexual Function Index
PERFECT	Power Endurance Repetitions Fast contractions Every Contraction Timed
MRS	Menopause Rating Scale
SF-12	Short-Form
PFDI-20	Pelvic Floor Distress Inventory
